# Knowledge of and attitude to eye disorders among pediatricians in North Jordan

**DOI:** 10.1016/j.amsu.2021.102430

**Published:** 2021-06-06

**Authors:** Laila T. Ababneh, Wadah Khriesat, Sarah Abu Dalu, Ranim J. Hanania, Bayan F. Ababneh, Nama’ A. Bany Amer, Thamer Jahmani

**Affiliations:** a Division of Ophthalmology, Department of Special Surgery, Faculty of Medicine, Jordan University of Science and Technology, Irbid, Jordan; b Division of Neonatology, Department of Pediatrics, Faculty of Medicine, Jordan University of Science and Technology, Irbid, Jordan; c Faculty of Medicine, Jordan University of Science and Technology, Higher Specialty Residency in Ophthalmology / King Abdullah University Hospital, Irbid, Jordan; d Division of Clinical Pharmacy, Faculty of Pharmacy, Jordan University of Science and Technology, Irbid, Jordan; e Department of Pediatrics, Residency in Pediatrics, Royal Medical Services, Jordan

**Keywords:** Refractive errors, Glaucoma, Retinoblastoma, Pediatrics

## Abstract

**Purpose:**

To assess the general knowledge of and attitude to various common eye conditions in children among pediatricians in north Jordan.

**Methods:**

This was a descriptive cross-sectional study. An exploratory questionnaire was developed by the research team to collect the necessary qualitative information.

**Results:**

In total, 48 pediatricians participated in the study. Around two-thirds performed eye examinations in children; however, only 10.4% (n = 5) carried out eye exams routinely as part of every child's health visit. The most common test done was assessment of red reflex, which was performed by only 60.4% (n = 29) of responders. Almost all participants (95.8%, n = 46) recognized the risk factors for retinopathy of prematurity (ROP). The majority of pediatricians (n = 46, 95.8%) would immediately refer a child with suspected glaucoma to an ophthalmologist. In regard to leukocoria, the majority recognized cataract (n = 38, 79.2%) and retinoblastoma (n = 40, 83.3%) as major causes of this condition. Almost all pediatricians (n = 46, 95.8%) would immediately refer a child with leukocoria to an ophthalmologist. On the subject of refractive errors, only 62.5% (n = 30) of physicians confirmed that refractive errors could occur in children of any age and that they may need glasses. Nonetheless, 70.8% (n = 34) stated that refractive errors could be a cause of squint in children. Amblyopia and underlying central causes of squint were a major concern for most responders with 68.8% (n = 33) and 75% (n = 36), respectively.

**Conclusion:**

Although the knowledge of and attitude to eye disease among pediatricians were at a satisfactory level, the degree of collaboration with ophthalmologists is limited. More workshops need to be held for pediatricians.

## Highlights

• Knowledge of eye disease among pediatricians were at a satisfactory level.
• The degree of collaboration with ophthalmologists is limited.
• More workshops need to be held for pediatricians.

## 1 Introduction

A global and comprehensive approach to evaluating patients in pediatric practice is essential to ensure the proper detection and diagnosis of the various pediatric disease entities, and to promote and maximize the well-being of children. According to the American Academy of Pediatrics, the American Association for Pediatric Ophthalmology and Strabismus, and the American Academy of Ophthalmology, ophthalmic examination should be performed at birth and at all child health visits [1]. Early detection and prompt treatment of ocular disorders in children are important in order to avoid lifelong visual impairment. Accordingly, pediatricians should be able to identify common visual problems and ocular structural abnormalities, notably retinopathy of prematurity (ROP), leukocoria, congenital glaucoma, congenital cataract, refractive errors, amblyopia, strabismus, and red eye.

## 2 Materials and methods

A descriptive cross-sectional study design was implemented to assess ophthalmic knowledge among pediatricians practicing in Irbid, north Jordan. The research team designed a questionnaire which met the research objective, ensured that respondents fully understood the questions and encouraged respondents to provide accurate, unbiased and complete information. The exploratory questionnaire was divided into two sections; the first section addressed the demographics of the participants; the demographic data included age, sex and years of practice; and the second section addressed the pediatricians’ knowledge of commonly encountered eye pathologies and presentations such as painful red eye, abnormal pupillary reflex, eye deviation, etc. and how it was approached in their daily practice.

The targeted population included pediatric consultants, specialists, and residents practicing in different sectors in northern Jordan. Hard copies of the questionnaires were distributed to the pediatric departments at King Abdullah University Hospital, Princess Rahma Pediatric Hospital and Prince Rashid Ben Al-Hasan Military Hospital. Participation in the study was completely voluntary and anonymous. A total of 48 participants completed the questionnaire (n = 48). Participants were asked to sign a written consent (Appendix 1) with a detailed explanation of the study. All information was treated confidentially and used for academic purposes only. The work was not funded and there was no conflict in interest.

For an overview of the consent and questionnaire, see Appendix 1.

## 3 Results

A total of 48 doctors participated in the study. The distribution of participants by sociodemographic status, practice duration, working status, working in an institution with an ophthalmology department, and participating in conferences on pediatric eye conditions is demonstrated in Table 1.

**Table 1:** Distribution of participants by sociodemographic status, duration of practicing, working status, working in institution with ophthalmology department, participating in conferences on children's eye conditions (n = 48).

Variable	
Age, mean (SD), years	32.13 (7.2)
Range, years	24–56
Sex, n (%)	
Male	22 (45.8%)
Female	26 (54.2%)
Total	48 (100%)
Length of participation as a pediatrician, mean (SD), years	5.06 (5.34)
Range, years	0.5–20
Working in institution with ophthalmology department, n (%)	
No	22 (45.8%)
Yes	26 (54.2%)
Total	48 (100%)
Working status, n (%)	
Pediatric resident	34 (70.8%)
General pediatrician	7 (14.6%)
Subspecialized pediatrician	7 (14.6%)
Total	48 (100%)
Participating in conferences on children's eye conditions, n (%)	
No	35 (72.9%)
Yes	13 (27.1%)
Total	48 (100%)

In total, 54.2% (n = 26) were female doctors while 45.8% (n = 22) were male doctors with age ranging from 24 to 56 years; mean age was 32.13 years. The mean years in practice as a pediatrician was 5.06 years; 54.2% (n = 26) had an ophthalmology department in their work institute. Despite the importance of basic knowledge among pediatricians with regard to eye diseases in children, the majority (n = 35, 72.9%) reported they had never attended a conference or a workshop concerning issues about various eye conditions in children.

Table 2 shows the distribution of participants by knowledge about causes, signs and symptoms, risk factors, and ways of management of specific eye problems.

**Table 2:** Distribution of participants by knowledge about causes, s/s, risk factors, and ways of management for specific eye problems (n = 48).

Variable	n (%)
When should an ophthalmologist see a child?	
No need if there is no complain	5 (10.4%)
Should have visual screening at least once before going to kindergarten	37 (77.1%)
I do not know	6 (12.5%)
Total	48 (100%)
Which of the following can cause painful red eye disease in children – more than one choice	
Conjunctivitis	45 (93.8%)
Allergy	33 (68.8%)
Uveitis	36 (75%)
Corneal abrasion/trauma	38 (79.2%)
Cataract	3 (6.3%)
Glaucoma	13 (27.1%)
Squint	0 (0%)
Which of the following can cause leukocoria (white pupil reflex) – more than one choice	
Cataract	38 (79.2%)
Glaucoma	9 (18.8%)
Retinoblastoma	40 (83.3%)
Advanced retinal disorder	25 (52.1%)
Leukocoria could be – more than one option	
Sight-threatening	32 (66.7%)
Life-threatening	18 (37.5%)
Normal variation between children	5 (10.4%)
Children of any age may have refractive errors and may need glasses	
True	30 (62.5%)
False	12 (25%)
I do not know	6 (12.5%)
Total	48 (100%)
Refractive errors can cause squint	
True	34 (70.8%)
False	6 (12.5%)
I do not know	8 (16.7%)
Total	48 (100%)
Which of the following give a clue that this child may have TRUE squint – more than one choice	
Eye deviation	42 (87.5%)
Face turning	27 (56.3%)
Anomalous head posture	23 (47.9%)
Epicanthal folds	2 (4.2%)
Wide nasal bridge	2 (4.2%)
What are the concerns about a child with squint – more than one choice	
Cosmetically not acceptable	10 (20.8%)
Amblyopia	33 (68.8%)
Underlying central cause	36 (75%)
Squint can be repaired by – more than one choice	
Glasses	37 (77.1%)
Surgical repair	40 (83.3%)
Spontaneously resolving	28 (58.3%)
Which of the following is a sign of congenital glaucoma – more than one choice	
Eye watering	17 (35.4%)
Leukocoria	16 (13.3%)
Large cornea	21 (43.8%)
Hazy cornea	31 (64.6%)
Red eye	10 (20.8%)
Which of the following may be a risk factor for ROP	
Birth weight < 1500 g	1 (2.1%)
Gestational age ≤ 32 weeks	1 (2.1%)
Premature baby with comorbidities	0 (0%)
All of the above	46 (95.8%)
Total	48 (100%)
Do you perform eye examinations in children?	
Yes	32 (66.7%)
No	16 (33.3%)
Total	48 (100%)
How often do you perform eye examinations?	
When caregiver reports child has eye problem	15 (31.3%)
At birth	9 (18.8%)
As a routine part of every child's exam	5 (10.4%)
Once a year	2 (4.2%)
What test do you do? – more than one choice	
Red reflex	29 (60.4%)
Visual acuity	13 (27.1%)
Funduscopic exam	10 (20.8%)
Extraocular muscle motility	15 (35.4%)
If no, why? – more than one choice	
Don't have enough time	5 (10.4%)
No equipment	9 (18.8%)
Don't know how to	6 (12.5%)
It's not relevant to my profession	6 (12.5%)
How do you manage a child with painful red eye?	
Refer immediately to ophthalmologist	20 (41.7%)
Give eye drops and refer immediately	6 (12.5%)
Give eye drops and if no improvement after 3 days refer to ophthalmologist	21 (43.8%)
Others	1 (2.1%)
Total	48 (100%)
How do you manage a child with leukocoria?	
Refer immediately to ophthalmologist	46 (95.8%)
Give eye drops	0 (0%)
Follow up and if no improvement refer to ophthalmologist	2 (4.2%)
Others	0 (0%)
Total	48 (100%)
How do you manage a child with squint?	
Refer immediately to ophthalmologist	29 (60.4%)
Give eye drops	1 (2.1%)
Follow up and if no improvement refer to ophthalmologist	3 (6.3%)
Brain imaging	15 (31.3%)
Others	0 (0%)
Total	48 (100%)
How do you manage a child with congenital glaucoma?	
Refer immediately to ophthalmologist	46 (95.8%)
Give eye drops	0 (0%)
Follow up and if no improvement refer to ophthalmologist	2 (4.2%)
Others	0 (0%)
Total	48 (100%)
When do you refer a premature baby for ROP screening?	
When discharged from NICU	10 (20.8%)
At 4–6 weeks after birth or at 32 weeks, whichever is later	36 (75%)
At birth	2 (4.2%)
Total	48 (100%)

NICU, neonatal intensive care unit; ROP, retinopathy of prematurity.

In answer to when an ophthalmologist should see a child, most participants (n = 37, 77.1%) knew that a child should be seen by an ophthalmologist for visual screening at least once before entering kindergarten.

Concerning questions about causes of painful red eye in children, 93.8% reported conjunctivitis as a cause, 79.2% reported corneal abrasion, 75% reported uveitis, and 68.8% reported allergy; however, glaucoma was the least mentioned with only 27.1% (n = 13). In terms of management, 41.7% of pediatricians would manage a painful red eye by immediately referring the patient to an ophthalmologist, 12.5% would give eye drops and then immediately refer to an ophthalmologist, while 43.8% would give the patient eye drops and if there was no improvement within 3 days, then they would proceed with referral.

Regarding leukocoria, the majority recognized cataract (n = 38, 79.2%) and retinoblastoma (n = 40, 83.3%) as major causes of this condition. Moreover, 52.1% chose massive retinal disorder as a cause. Only a minority (n = 9, 18.8%) believed that glaucoma could be a cause. In terms of the implications of leukocoria, that it could be sight-threatening was known by 66.7% (n = 32) of participants. Furthermore, only 37.5% (n = 18) of participants reported that it could also be life-threatening, and 10.4% (n = 5) believed that it could be a normal variation among children, which is false. Almost all pediatricians (n = 46, 95.8%) would immediately refer a child with leukocoria to an ophthalmologist, and only 4.2% would do a follow up and if there was no improvement, then they would refer the child.

On the subject of refractive errors, only 62.5% (n = 30) of physicians confirmed that refractive errors could occur in children of any age and that they may need glasses. The rest wrongly believed they did not occur in children (n = 12, 25%) or they did not know (n = 6, 12.5%). In addition, 70.8% stated that refractive errors could be a cause of squint in children. The participants’ answers on signs of a true squint were as follows: 87.5% reported eye deviation, 56.3% reported face turning, and 47.3% reported anomalous head posture. Epicanthal folds and wide nasal bridge were both mentioned by 4.2% of physicians.

Amblyopia and underlying central causes of squint were a major concern for most physicians with 68.8% and 75%, respectively, while cosmetic concern was only mentioned by 20.8%. In relation to management of squint, 83.3% chose surgical repair and 77.1% chose glasses. However, more than half reported that it could also resolve spontaneously. In addition, 60.4% of pediatricians would immediately refer a child with squint to an ophthalmologist and 31.3% would order brain imaging, while 6.3% would follow up with the child and refer if no improvement. Only 2.1% would prescribe eye drops.

Concerning congenital glaucoma and its signs, 64.6% reported hazy cornea, 43.8% reported large cornea and 35.4% reported eye watering. Red eye was chosen by 20.8% and leukocoria by 13.3% of physicians. The majority of pediatricians (n = 46, 95.8%) would immediately refer a child with suspected glaucoma to an ophthalmologist, only 4.2% chose to follow up with the child and refer if there was no improvement. No participant chose to give eye drops for management.

Almost all participants recognized the risk factors for retinopathy of prematurity (ROP) as 95.8% (n = 46) chose birth weight less than 1500 g, gestational age of less than or equal to 32 weeks, and a premature baby with comorbidities [2,3]. Concerning ROP screening, 75% of pediatricians would refer a premature baby for ROP exam 4–6 weeks after birth or at 32 weeks, whichever is later, whereas 20.8% would refer a baby for ROP examination at the time of discharge from the NICU, and 4.2% chose that the ROP examination should be done at birth.

On the topic of pediatric practices in the sample group, around two-thirds performed eye examinations in children; however, 31.3% would perform an eye exam when the child's care giver reported a problem, and only 10.4% would do the eye exam routinely as part of every child's health visit. Moreover, 4.2% would do the eye exam once a year and 18.8% would do the examination at birth.

Among our participating physicians, 60.4% assessed red reflex, 35.4% assessed extraocular muscle motility, and 27.1% assessed visual acuity, while 20.8% performed fundoscopic examinations.

The pediatricians who did not perform eye examinations were asked about their reasons, and 18.8% stated that they did not have the necessary equipment, 10.4% did not have sufficient time, 12.5% did not know how to do the examination, and 12.5% believed it was not relevant to their profession.

## 4 Discussion

It is crucial that common eye conditions in children are properly diagnosed and managed by pediatricians or primary care physicians. Some eye diseases could be sight-threatening or even life-threatening, therefore, we decided to conduct this study to assess the ophthalmic knowledge and practices of the Jordanian pediatric body in Irbid, northern Jordan.

According to the American Academy of Pediatrics, vision screening from birth through adolescence is recommended, with visual acuity testing and binocular screening to begin at 3 years of age [4]. In addition, the American Association for Pediatric Ophthalmology and Strabismus recommends that screening should be at least once from the age of 12–36 months [5]. In our study, 77.1% of pediatricians thought that children should be seen at least once before entering kindergarten, while 10.4% believed that they should be seen only if there was a complaint, and 12.5% did not know when children should be screened. The problem lies in the fact that many of the childhood eye diseases are silent. Also, children may not be able to complain or communicate well about vision problems until later in life. Ophthalmic screening and eye examination are very important in children, therefore, it is highly recommended that the pediatric departments in Jordanian teaching hospitals invest in training their staff accordingly.

Knowledge of the participants in relation to the causes of painful red eye disease was very good. The most common causes reported were conjunctivitis (93.8%) and corneal abrasion/trauma (79.2%), followed by uveitis and allergy. Our results are comparable with a similar study performed in Kenya, where the Kenyan study found that trauma and conjunctivitis were also the most common causes chosen by responders for a painful red eye [6].

Regarding the causes of leukocoria, 83.3% of responders mentioned retinoblastoma, which is a much higher percentage in comparison to a study conducted in Brazil by Manica et al. where retinoblastoma was mentioned by only 37% of responders [7]. Other causes mentioned included cataract (79.2%) and advanced retinal disorder (52.1%). Glaucoma, which is not a cause of leukocoria, was mentioned by 18.8% of responders [8]. The response was very good, as retinoblastoma, the most serious and life-threatening cause, was mentioned by the majority. Furthermore, cataract, which is the most common cause of leukocoria, was also mentioned by the majority of responders. Both eye diseases can cause lifelong visual impairment if not diagnosed and treated promptly; 66.7% of responders stated that retinoblastoma/leukocoria is a sight-threatening disease, while 37.2% stated that it could be life-threatening too. Almost all pediatricians (n = 46, 95.8%) would immediately refer a child with leukocoria to an ophthalmologist. In another study on retinoblastoma awareness in Jordan, only 2% of pediatricians scored a proficiency grade in the questionnaire and only 63% of pediatricians knew that retinoblastoma required urgent management [9].

Concerning the knowledge of refractive errors among children, only 62.5% of our responders knew that children may have refractive errors. However, 70.8% of them admitted that refractive errors are a major cause of squint. In comparison, in the study in Kenya, almost all of their responders (98.4%) were aware that refractive errors occurred in childhood [6]. The result from our participants is unsatisfactory. It is crucial to detect refractive errors as early as possible to avoid complications such as amblyopia and refractive squint. In a study conducted in China on more than 5000 children, the association between refractive errors and the prevalence of concomitant esotropia and concomitant exotropia was significant, which should be considered when managing childhood refractive errors [10].

Squint or eye deviation was recognized as a cardinal sign by most pediatricians (87.5%). Other signs such as face turning and anomalous head posture were also mentioned [11]. Only 4.2% wrongly stated that the signs for pseudosquint, which are wide nasal bridge and epicanthic folds, were clinical signs for true squint [11]. Their main concern for children with squint was an underlying central cause which was mentioned by 75% of participants. This was followed by amblyopia with 68.8% and only 20.8% mentioned cosmetic reasons as a concern. In terms of management, 60.4% of responders said they would refer children with squint to ophthalmologists, while 31.3% would recommend brain imaging. Although squint was known by participants to be a condition which was treatable by either surgical repair (83.3%) or glasses (77.1%), more than half (58.3%) continued to wrongly answer that it could resolve on its own. It was reassuring that the majority could identify the signs of strabismus and would refer to ophthalmologists for further evaluation and management. On the other hand, we encourage a joint effort between pediatricians and ophthalmologists in Jordan to work together and stay updated with the Jordanian ophthalmic guidelines for screening, examination and management of strabismus.

Regarding congenital glaucoma, 64.4% chose hazy cornea as a clinical sign, while 43.8% chose a large cornea, and 35.4% chose eye watering. According to this result, many children with congenital glaucoma could be missed. Moreover, 13.3% reported leukocoria as a clinical sign and 20.8% reported a red eye, which is incorrect. The result is not satisfactory and more awareness of this ocular disease should be brought to the attention of Jordanian pediatricians in Irbid.

Risk factors leading to retinopathy of prematurity (ROP) were recognized by most responders (95.8%) in this study. This is a large percentage compared to a study conducted in India, whereby 42.2% were not aware of the risk factors [12]. Early detection and management of ROP decrease morbidity among premature babies. A total of 75% of participants would refer a baby for ROP screening 4–6 weeks after birth or at the age of 32 weeks, whichever is later. The results are satisfactory in comparison to a study done in the West Bank by Akkawi et al., where 41.4% did not know exactly when ROP screening should be started [13].

Assessment of the participants' practices showed a satisfactory level overall. Among participants, 66.7% performed eye examinations on children; however, only 10.4% performed this routinely as part of every child's health visit. This percentage is low in comparison to the Kenyan study, where 43.52% of Kenyan pediatricians performed eye exams in their routine examinations [6].

In relation to eye exams and testing, 60.4% assessed red reflex, 27.1% checked visual acuity, and 20.8% performed fundus exams. This is in contrast to a study in the United States which included 576 pediatricians and where 95% assessed red reflex, 92% checked visual acuity, and 65% performed fundus exams [14]. Examinations done by our participants are deficient in comparison to US pediatricians. Red reflex is part of the pediatric general examination and when around 40% of pediatricians do not perform it, this will lead to missing many children with certain eye conditions and increase morbidity among them [[15], [16], [17]]. Moreover, one-third (33.3%) of pediatricians in our study did not perform eye exams, attributing this to different causes. For instance, some did not have sufficient time, equipment was unavailable, or they did not know how to do it, and some believed that it was not relevant to their profession.

According to our study, most pediatricians would refer a child with an eye problem to an ophthalmologist. When a child presented with painful red eye, 41.7% would proceed with referral immediately and 43.8% would prescribe eye drops then refer the child. This practice is similar to the practice of general practitioners in the US where 55% prescribed chloramphenicol ointment whenever they faced a patient with allergic or bacterial conjunctivitis, besides blepharitis and meibomian cysts, where all of these conditions may cause a red eye [14].

## 5 Conclusion

Although the knowledge of and attitude to eye diseases among pediatricians in northern Jordan were at a satisfactory level, the degree of collaboration with ophthalmologists is limited. More workshops need to be held for pediatricians.

Our work was conducted in accordance with STROCSS criteria [18].

## Ethical approval

Institutional approval was obtained from the Institutional Review Board at Jordan University of Science and Technology.

## Sources of funding

No funding.

## Author contribution

All authors contributed significantly and in agreement with the content of the article. All authors were involved in project design, data collection, analysis, statistical analysis, data interpretation and writing the manuscript. All authors presented substantial contributions to the article and participated of correction and final approval of the version to be submitted.

## Consent

Informed consent was obtained from each patient.

## Registration of research studies

researchregistry6754

https://www.researchregistry.com/browse-the-registry#home/?view_2_search=6754&view_2_page=1.

## Guarantor

Laila Ababneh.

## Provenance and peer review

Not commissioned, externally peer-reviewed.
