# Supplementary material for: Knowledge of and attitude to eye disorders among pediatricians in North Jordan
Source: Ann Med Surg (Lond). 2021 Jun 6;67:102430. doi: 10.1016/j.amsu.2021.102430 (PMC8209081; doi:10.1016/j.amsu.2021.102430)
Supplement: Multimedia component 2 [file mmc2.docx]

Appendix 1. An overview of the consent and questionnaire

| Dear colleagues,  We are conducting a study to assess the general knowledge about various eye conditions in children among Jordanian pediatricians.  We kindly ask you to fill this brief questionnaire. The information gathered will be confidential and used only for academic purposes, and to improve health services in our country.  Please feel free to participate in this study or to decline. If you are willing to participate, you are kindly requested to append your signature below, Thank you for your cooperation.  Dr. Laila Taha Ababneh – Assistant Professor, pediatric ophthalmologist.  Prof. Waddah Khreisat – Professor of pediatrics and neonatology.  Dr. Sarah Abu Dalu – Ophthalmology resident.  Dr. Ranim J. Hanania – Ophthalmology resident.  Declaration  I have read and understood the above explanation, and I am willing to participate in the study voluntarily.  Signature:  Date: |
| --- |

| PART ONE: |
| --- |
| 1. Age ____________ 2. Gender 3. Male 4. Female 5. How long have you been practicing as a pediatrician? _________ 6. You are currently working as 7. Pediatric resident 8. General pediatricians 9. Subspecialized pediatrician 10. Do you have in your working institute an ophthalmology department? 11. Yes 12. No 13. Have you ever participated in a workshop/conference concerning various eye conditions in children? 14. yes 15. No |
| PART TWO: |
| 1. When an ophthalmologist should see a child? 2. No need if there is no complaint 3. should have visual screening at least once before going to kindergarten 4. I don’t know 5. Which of the following can cause red painful eye disease in children - more than one option could be applicable 6. conjunctivitis 7. allergy 8. uveitis 9. corneal abrasion/trauma 10. cataract 11. glaucoma 12. squint 13. Which of the following can cause leukocoria (white pupil reflex ) – more than one option could be applicable 14. Cataract 15. Glaucoma 16. Retinoblastoma 17. Advance retinal disorder 18. Leukocoria could be - more than one option could be applicable 19. Sight threatening 20. Life threatening 21. Normal variation between children 22. Children of any age may have refractive errors and may need glasses 23. True 24. False 25. I don’t know 26. Refractive errors can cause squint 27. True 28. False 29. I don’t know 30. Which of the following gives a clue that a child may have TRUE squint - more than one option could be applicable 31. Eye deviation 32. Face turn 33. Anomalous head posture 34. Epicanthal folds 35. Wide nasal bridge 36. What are the concerns about a child with squint-- more than one option could be applicable 37. Cosmetically not acceptable 38. Amblyopia 39. Underlying central cause 40. Squint can be repaired by - more than one option could be applicable 41. Glasses 42. Surgical repair 43. Spontaneously resolving as child grows 44. Which of the following is a Sign of congenital glaucoma -- more than one option could be applicable 45. Watering 46. leukocoria 47. Large cornea 48. Hazy cornea 49. Red eye 50. Which of the following may be a Risk factors of ROP ( retinopathy of prematurity ) 51. Birth weight < 1500 gm 52. GA < / = 32 weeks 53. Premature baby with comorbidities 54. All of the above 55. Do you perform eye examination in children? 56. YES 57. NO --- if no please move to question 15 58. How often do you do eye examination? 59. when caregiver reports child has eye problem 60. at birth 61. as a routine part of every child’s examination 62. Once a year 63. What test do you do? -more than one option could be applicable 64. Red reflex 65. Visual acuity 66. Fundoscopic examination 67. Extraocular muscles motility 68. If no, why? - more than one option could be applicable 69. Don’t have enough time 70. No equipment 71. Don’t know how to 72. It’s not relevant to my profession 73. How do you manage a child with painful red eye 74. Refer immediately to ophthalmologist 75. Give eye drops and refer immediately 76. Give eye drops and if no improvement after 3 days refer to ophthalmologist 77. Others , indicate please ___________________________________ 78. How do you manage a child with leukocoria? 79. Refer to ophthalmologist immediately 80. Give eye drops , specify the eye drop ______ 81. Follow up and if no improvement refer to ophthalmologist 82. Others , indicate please ___________________________________ 83. How do you manage a child with squint? 84. Refer to ophthalmologist immediately 85. Give eye drops , specify the eye drop _____ 86. Follow up and if no improvement refer to ophthalmologist 87. Brain imaging 88. Others , indicate please ___________________________________ 89. How do you manage a child with congenital glaucoma ? 90. Refer to ophthalmologist immediately 91. Give eye drops , specify the eye drop ___________________ 92. Follow up and if no improvement refer to ophthalmologist 93. Others , indicate please ___________________________________ 94. When you will refer a premature baby for ROP screening? 95. When discharge from NICU 96. At 4-6 weeks after birth or at 32 weeks which ever later 97. At birth |
